# Benefits of physical activity on cognitive function in patients with neurocognitive disorders: A systematic review

**DOI:** 10.1016/j.tjfa.2025.100069

**Published:** 2025-09-04

**Authors:** Théodore Decaix, Claire Bonnin, Karl Götze, Véronique François, Camille Petit, Clémentine Rivière, Sandrine Greffard, Emmanuel Cognat, Jacques Hugon, Claire Paquet, Louise Sindzingre, Matthieu Lilamand

**Affiliations:** a Geriatrics Department, Fernand Widal Lariboisière University Hospital, GHU APHP.Nord, 75010 Paris, France; b Paris-Cité University, Inserm U1144, Paris, France; c Department of Geriatrics, Bichat Hospital (GHU AP-HP.Nord, Paris) Université Paris-Cité, 75018 Paris, France; d Cognitive Neurology Center, Fernand Widal Lariboisière University Hospital, GHU APHP.Nord, 75010 Paris, France

**Keywords:** Cognitive decline, Cognition, Neurodegenerative disease, Prevention, Physical exercise

## Abstract

Neurocognitive disorders, particularly in older adults, significantly affect functional abilities and global health. Physical activity has emerged as a potential non-pharmacological intervention to improve cognitive performance in patients with neurodegenerative diseases. This review specifically addressed the issue of tailored physical activity interventions for individuals with various neurocognitive disorders.

This literature review analyzed studies investigating the effects of physical activity on cognitive function in patients with Alzheimer’s disease (AD), vascular cognitive impairment, Parkinson’s disease, and Lewy body dementia. The studies were evaluated for methodological rigor, participant characteristics, types of physical activities, and cognitive outcomes.

Of the 21 studies reviewed, 14 reported beneficial effects of physical exercise on cognitive function, particularly with aerobic activities. While most studies observed improvements in cognitive performance and physical functional capacity, results were inconsistent, and effect sizes were modest. Mechanisms proposed for cognitive improvement in AD included reductions in β-amyloid and tau protein burdens, improved glucose metabolism, and enhanced brain-derived neurotrophic factor levels. Specific improvements were noted in Parkinson’s disease, with evidence suggesting mediation through dopamine pathways. Despite evidence of short-term benefits, significant variability exists among studies, highlighting the need for individualized exercise programs tailored to specific neurocognitive conditions.

Physical activity stands as a cornerstone non-pharmacological intervention, essential for supporting cognitive health in individuals with neurodegenerative diseases. Further research is necessary to establish long-term effects and optimal exercise modalities, along with standardized evaluation criteria to assess the cognitive impacts of physical activity reliably.

## 1 Introduction

The rising prevalence of neurocognitive disorders, driven by an aging population, has substantial implications for morbidity and mortality. Despite advances in pharmacological research, a crucial element of primary and secondary prevention includes non-pharmacological measures, among which tailored physical activity plays a central role. Exercise training has been suggested as a cornerstone, improving both cognitive and physical functions in healthy older adults [[1], [2], [3]]. Greater physical activity is associated with reduced insulin levels and body mass index, supporting healthier body composition in older adults with normal cognition. Mid-life obesity has been linked to an increased risk of cognitive impairment in later years, while sarcopenia—a decline in muscle mass and strength—has also been correlated with the incidence of neurocognitive disorders [[4], [5], [6]]. Furthermore, physical activity mitigates chronic inflammation and oxidative stress, both of which contribute to the risk of cognitive decline. Better cardiovascular and metabolic health through regular physical activity can simultaneously lower the risk of neurodegeneration, thereby reducing the likelihood of cognitive decline and dementia [[7], [8], [9]]. Literature indicates that physical activity particularly benefits executive functioning and memory in Mild Cognitive Impairment (MCI), independent functioning in MCI and dementia, and psychological health in dementia [10]. Processing speed might also improve for patients with dementia thanks to exercise [11,12]. Furthermore, physical activity may positively impact non-cognitive outcomes such as disability, falls, and neuropsychiatric symptoms in people with dementia [13]. The benefits of physical activity have been suggested in Alzheimer Disease (AD), with research such as Jia et al.'s meta-analysis showing that physical activity and exercise could improve cognition in older patients with AD [14]. Two umbrella reviews have also highlighted the positive effects of physical activity in both individuals with MCI and dementia, including AD dementia [13,15]. Finally, a recent review and meta-analysis suggested that physically active subjects had a 20 % risk reduction of incidence of AD and associated disorders compared to inactive individuals [16]. Besides, in patients with AD, physical activity was associated with significant cognitive and functional benefits [17]. Nevertheless, a critical review of published evidence has toned down the actual effect of exercise on cognition reported in previous meta-analytic reviews, which may have been overestimated. In general, the available causal evidence from randomized controlled trials (RCT) on the link between exercise and cognition is far from conclusive [18]. Similarly, in another study supervised exercise in patients with dementia was associated with slight worsening in cognitive performance after one year, questioning the systematic value of such intervention [19]. While there is substantial evidence regarding the global effects of physical activity on cognitive performance, considerable heterogeneity exists regarding cognitive diagnosis and types of exercise training. In older adults with all-cause dementia, recruited from daycare and residential care facilities, neither low nor high-intensity training was effective on cognitive performance after 36-week follow-up [20]. Differences related to the causal pathology (AD, vascular cognitive impairment (VCI), Parkinson’s disease (PD), Lewy body disease (LBD) could explain part of the heterogeneity of these interventions. A critical review of the literature focusing on the pathologies responsible for cognitive disorders could help better define the benefits and limitations of tailored physical activity in older adults with cognitive disorders and highlight the interventions that have proven effective. This review aims to find evidence of the associations between physical activity and cognitive performance, considering established neurocognitive diagnoses and types of exercise training.

## 2 Methods

### 2.1 Literature search strategy

We conducted a systematic search following the Preferred Reporting Items for Systematic Reviews and Meta-Analyses (PRISMA) guidelines [21]. The search aimed to identify studies looking at the link between physical activity and cognitive function in patients with an identified neurocognitive disease. We identified all published articles between January 2015 and July 2024 using the PubMed interface (Medline database), Web of Science and the Cochrane Library. The keywords used were the Medical Subject Heading (MeSH) terms “physical activity” or “exercise” and “cognition” or “cognitive performance”. The last search was carried out on July 30, 2024.

### 2.2 Eligibility

Experimental studies, randomized and non-randomized control trials (RCT and NRCT) and crossover studies, were eligible. On the other hand, meta-analyses, reviews, observational studies, letters were dismissed. The Population, Intervention, Comparison and Outcome (PICO) characteristics for eligibility were: (P) - Population: patients with an identified neurocognitive disorder such AD, VCI, PD or LBD aged 60 years and older; (I) - Intervention: regular exercise program with a minimum duration of two weeks and involving aerobic exercise, resistance exercise or other training program; (C) Comparison with an active control group (in which participants completed a different exercise program or an alternative activity) or a passive control group (participants did not complete any exercise program); (O) Outcomes: Measurement of global cognitive functioning or specific cognitive domains, such as executive functions, attention, and memory. Our objective was to examine the relationship between physical activity and cognitive change in various groups of cognitively impaired older adults due to neurodegenerative or vascular diseases. Consequently, we only included controlled studies with an interventional arm involving physical activity, as well as studies with participants diagnosed with cognitive disorders of known etiology. Studies in which combined exercise programs with cognitive training, or any other intervention, were also excluded. Studies published in languages other than English and French were excluded.

### 2.3 Selection process

After elimination of duplicates, the titles and abstracts were screened by two independent researchers (ML, CB). Full text articles that were deemed ineligible were excluded. We subsequently reviewed all papers cited in the selected articles and included additional references based on their originality and relevance to the review's scope. Exclusion reasons were documented at each stage, and any discrepancies in data extraction were resolved through researcher discussions.

### 2.4 Data extraction

Data extraction was carried out by the same two authors (ML, CB) using a standardized extraction form and working independently. Both collected data from the studies, organizing the information into a standardized table with consistent columns. These columns included study design (RCT, NRCT and crossover studies), sample size, population, cognitive diagnosis, mean age, sex, Mini-mental State Examination (MMSE) or other cognitive scores at baseline, Unified Parkinson’s Disease Rating Scale (UPDRS) motor subsection and Hoehn and Yahr stage for patients with PD and LBD, exercise modality, session length, frequency, progression and program duration, cognitive outcomes, other key findings including potential side effects of the intervention. Effect sizes and mean changes in cognitive function (95 % Confidence Interval) before and after the physical activity intervention were reported when available for significant results; otherwise, they were calculated.

## 3 Results

### 3.1 Study selection

**Fig. 1:**
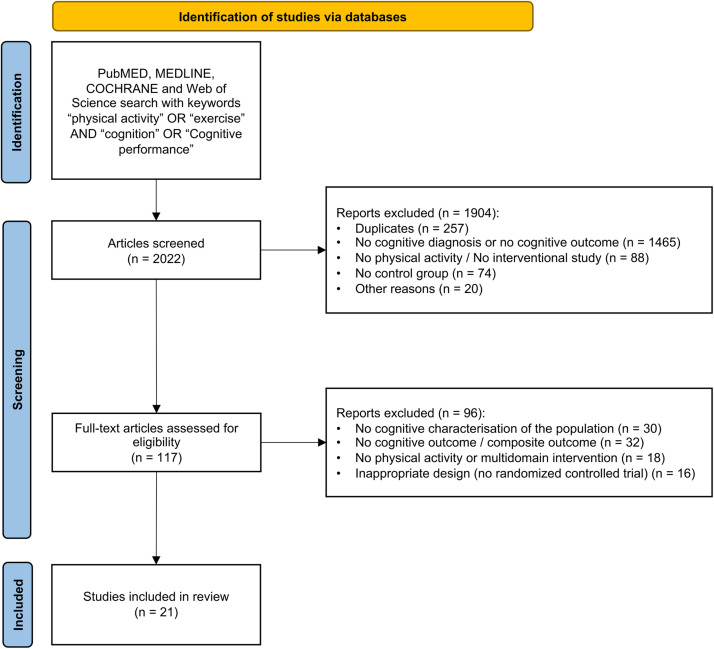
Study flowchart.

A total of 2022 articles were identified in the original search. After an initial screening, 117 citations underwent a full-text review by the two reviewers (ML, CB). Of these, 30 articles did not present a cognitive characterisation of the population, 32 articles did not assess change in cognition, 18 articles did not comprise an interventional group undergoing physical activity or comprise cognitive exercise, and 16 articles were excluded due to the lack of a diagnosis, because the patients were under 60 years of age or because of an inappropriate design. Ultimately, 21 studies [[22], [23], [24], [25], [26], [27], [28], [29], [30], [31], [32], [33], [34], [35], [36], [37], [38], [39], [40], [41], [42]] were included in this review: 7 articles involved community-dwelling patients with AD, 3 with VCI, 10 with PD and 1 with LBD and PD dementia. A detailed report of the selection process is provided in the Flowchart in Fig. 1.

### 3.2 Baseline characteristics

The mean age of the participants ranged from 62.3 ± 5.7 to 80.4 ± 6.5 years old. Three studies included a majority of women [22,31,33], 13 a majority of men [24,[27], [28], [29],^32^,[34], [35], [36], [37], [38], [39], [40],^42^] and 5 an equal proportion of men and women [23,25,26,30,41]. The duration of the interventions varied from 1 month to 18 months [29,35]. Seven studies compared a physical intervention with a passive control group, two studies with a health education group, nine with usual care including conventional physical therapy or exercise program, and three with an alternative activity. One study was a crossover trial [42], one study a quasi-randomized trial [38] and one a quasi-experimental parallel group study [33]. Numerous cognitive function assessment tools were used (**See details in** Tables 1**,** 2 **and** 3). The studies were conducted across various regions: six in North America [26,28,30,31,34,37], two in South America [36,38], four in Asia [22,32,39,40], seven in Europe [[23], [24], [25], [26], [27],^35^,^41^] and two in Australia [33,42].

**Table 1:** Characteristics of studies regarding the relationship between physical activity and cognitive performance, considering types of exercise training, in patients with Alzheimer’s disease.

Study first author, year	Study design	Sample size	Population	Mean age ± SD (years)	Female (%)	Mean global cognitive assessment at baseline ± SD	Exercise Modality vs. Control Group	S session length F frequency per week D program duration	Cognitive assessment scale	Post-Intervention Outcome Differences: Intervention vs. Control (p-value)	Effect size
Lok, 2023 [23]	RCT	85	AD	73.3 ± 4.4	51	MMSE 23.4 ± 0.5	Musical exercise and walking vs. control group	S: 3 mths F: 5 D: 30–40 min	MMSE	Mean change (95 % CI): 2.14 (1.85 – 2.42) vs. - 0.23 (- 0.45 to - 0.10) (*p* = 0.048)	Cohen’s *d* 19.64

Holthoff, 2015 [25]	RCT	30	AD	72.4 ± 4.3	50	MMSE 20.6 ± 6.5	Home-based physical activity training vs. health education group	S: 12 wks F: 3 D: 30 min	MMSE Reaction time Verbal fluency	Mean change (95 % CI): No difference - 0.03 (- 0.04 to – 0.02) vs. 0 (- 0.008 to – 0.008) (*p* < 0.05) 0.55 (0.02 – 1.07) vs. - 1.87 (−2.89 to – 1.35) (*p* < 0.05)	- Cohen’s *d* 2.12 Cohen’s *d* 2.57

Öhman, 2016 [27]	RCT	210	AD	78.1 ± 5.2	39	MMSE 18	Home-based exercise vs. group-based exercise vs. control group	S: 52 wks F: 2 D: 60 min	MMSE CDT Verbal fluency	Mean change (95 % CI): No difference 0.54 (0.17 – 1.00) vs. 0.06 (- 0.38 – 0.49) vs. - 0.14 (- 0.57 – 0.31) (*p* < 0.0001) - 0.95 (- 1.62 to – 0.20) vs. - 0.76 (- 1.37 to – 0.11) vs. - 0.90 (−1.54 to – 0.24) (*p* = 0.02)	- Cohen’s *d* Group 1 vs. 2: 1.33 Group 1 vs. 3: 1.89 Group 2 vs. 3: 0.55 Cohen’s *d* Group 1 vs. 2: 0.58 Group 1 vs. 3: 0.15 Group 2 vs. 3: 0.43

Lam, 2022 [22]	RCT	376	AD	80.4 ± 6.5	80	MMSE 21.2 ± 2.9	Physical exercise group vs. Working memory training group vs. Physical exercise-Working memory group vs. health education group	S: 6 wks F: 2 D: 45 min	ADAS-Cog CDR-SOB Delay recall	No difference	–

Hoffmann, 2016 [24]	RCT	200	AD	70.5 ± 7.4	43	MMSE 24 ± 3.6	Moderate-to-high intensity aerobic exercise vs. control group	S: 16 wks F: 3 D : 60 min	SDMT ADAS-Cog Stroop test MMSE Verbal fluency	No difference	–

Morris, 2017 [26]	RCT	76	AD	72.9 ± 7.7	51	MMSE 25.4 ± 3.2	Aerobic exercise vs. non-aerobic stretching and toning control	S : 26 wks F : 1 D : 150 min	Logical Memory FCSRT BNT Digit Span Verbal fluency D-KEFS CCFCS LNS Stroop test	No difference	–

Yu, 2021 [28]	RCT	96	AD	77.4 ± 6.8	45	MMSE 21.4 ± 3.3	Cycling vs. stretching	S: 6 mths F: 3 D: 20–50 min	ADAS-Cog LM, HVLT-R TMT A and B EXIT-25, CDT Digit Span Verbal fluency BNT	No difference	-

AD: Alzheimer’s disease; ADAS-Cog: Alzheimer’s Disease Assessment Scale–Cognitive subscale; BNT: Boston Naming Test; CDR-SOB: Clinical Dementia Rating sum of boxes; CDT: Clock Drawing Test; D-KEFS CCFCS: D-KEFS Confirmed Correct and Free Card Sorting; EXIT-25: Executive Interview-25; FCSRT: Free and Cued Selective Reminding Test; HVLT-R: Hopkins Verbal Learning Test-Revised; LM: Logical Memory; LNS: Letter Number Sequencing; MMSE: Mini-mental State Exam score; RCT: randomized control trials; SD: standard deviation; SDMT: Symbol Digit Modalities Test; TMT: Trail Making Test.

**Table 2:** Characteristics of studies regarding the relationship between physical activity and cognitive performance, considering types of exercise training, in vascular cognitive impairment patients.

Study first author, year	Study design	Sample size	Population	Mean age ± SD (years)	Female (%)	Mean global cognitive assessment at baseline ± SD	Exercise Modality vs. Control Group	S session length F frequency per week D program duration	Cognitive assessment scale	Post-Intervention Outcome Differences: Intervention vs. Control (p-value)	Effect size
Ihle-Hansen, 2019 [29]	single-blinded RCT	380	Stroke	71.7 ± 11.3	40	MMSE 28 ± 0.2	Regular individualized coaching vs. standard care	S: 18 mths F: 7/2–3 D: 30/45–60 min	MMSE TMT A, B	No difference	–

Barha, 2017 [30]	single-blinded RCT	71	Subcortical ischemic VCI	73.8 ± 7.5	52	MMSE 26.7 ± 2.4	Aerobic Training (walking) vs. Usual care plus education group	S: 6 mths F: 3 D: 60 min	Stroop test Digit span TMT A, B	Change mean (SD): No difference No difference Improved according to the sex (Female > Male): F(1.47) = 4.196 (*p* < 0.05)	- - ηp^2^ = 0.082

Hsu, 2018 [31]	RCT	21	Mild subcortical ischaemic VCI	72 ± 8.8	61	MMSE 27.3 ± 1.9	Usual care vs. Walking aerobic training	S: 6 mths F: 3 D: 60 min	Eriksen flanker task	Change mean (SD): - 40.9 ± 17.2 ms vs. - 136.7 ± 18.1 ms (*p* < 0.05)	Cohen’s *d* 5.42

MMSE: Mini-mental State Examination ; RCT: randomized control trials ; SD: standard deviation ; TMT: Trail Making Test ; VCI: vascular cognitive impairment.

**Table 3:** Characteristics of studies regarding the relationship between physical activity and cognitive performance, considering types of exercise training, in PD and LBD patients.

Study first author, year	Study design	Sample size	Population	Mean age ± SD (years)	Female (%)	Mean global cognitive assessment at baseline ± SD	Exercise Modality vs. Control group	S session length F frequency per week D program duration	Cognitive assessment scale	Post-Intervention Outcome Differences: Intervention vs. Control (p-value)	Effect size
Liu, 2022 [32]	Single-blind RCT	28	PD	67.9 ± 6.5	42	MMSE 28.7 ± 2.0	Square-stepping and exercise* vs. Conventional physical therapy	S: 8 wks F: 2 D: 60 min	TMT A TMT B Digit span task MoCA	No difference	

Kalyani, 2019 [33]	Quasi-experimental parallel group study	38	PD	65.9 ± 9.8	60	ACE 93.2 ± 3.8	Dance vs. Control group	S: 12 wks F: 2 D: 60 min	NIH Toolbox: Improved Auditory verbal learning test TMT A TMT B	Mean change (95 % CI): 6.97 (4.44 – 9.50) vs. 3.21 (0.91 – 5.51) (*p* = 0.04) No difference - 17.71 (- 31.31 to - 4.11) vs. 5.76 (6.57 – 18.11) (*p* = 0.02)	Cohen’s *d* 0.81 - Cohen’s *d* 0.93

Silveira, 2018 [34]	Non-blinded RCT	76	PD	69.3 ± 8.7	28	MoCA 25.2 ± 4.5	Aerobic exercise cycling vs. goal-based exercise⁎⁎ vs. control group (continue with their regular activities)	S : 12 wks F: 3 D: 60 min	Digit Span Corsi Block test TMT A, B Stroop test CVLT, Rey-O Fluency tasks BNT, IPBLOT	No difference	

Picelli, 2016 [35]	Single-blind, RCT	17	PD	71.4 ± 8.2	25	MoCA 23.5	Treadmill training vs. no physical training	S: 1 mths F: 3 D: 45 min	FAB-it MoCA TMT A TMT B MI test	Change mean (95 % CI): 0.011 (1.16 – 3.95) vs. 0.705 (−1.09 – 1.09) (*p* = 0.005) No difference 0.018 (- 37.32 to – 3.35) vs. 0.735 (- 10.14 to – 12.64) (*p* = 0.027) 0.008 (- 79.75 to – 21.14) vs. 0.345 (- 67.68 to – 39.43) (*p* = 0.009) 0.010 (1.53 – 3.58) vs. 1.0 (- 1.84 – 1.84) (*p* = 0.014)	Cohen’s *d* 0.93 - Cohen’s *d* - 0.51 Cohen’s *d* - 0.33 Cohen’s *d* 0.57

Silva-Batista, 2020 [36]	Single-blinded RCT	40	freezers PD	65.7 ± 9.7	34	MMSE 25.6 ± 1.9	Adapted resistance training with instability vs. traditional motor rehabilitation	S: 12 wks F: 3 D: 80–90 min	Stroop test	No difference	–

Li, 2024 [39]	RCT	95	PD	62.3 ± 5.7	38	MMSE 28.9 ± 1.37	Tai Chi vs. Brisk walking vs. Control group	S: 12 mths F: 2 D: 60 min	PDCRS	Mean change (95 % CI): Tai Chi vs. Control: 8.8 (2.58 – 15.02) vs. – 4.4 (- 9.98 – 1.18) (*p* = 0.045) Tai Chi vs. Brisk walking: No difference	Cohen’s *d* 4.38

Hashimoto, 2015 [40]	Quasi-randomized pilot trial	46	PD	66.8 ± 8.6	26	MMSE 28.3 ± 2	Dance group vs. PD exercise group vs. non-intervention group	S: 12 wks F: 1 D: 60 min	Frontal Assessment Battery (FAB) MRT, Mental Rotation Task	Interaction between the 3 groups: F(2.42) = 4.3 (*p* = 0.02) F(2.42) = 11.4 (*p* < 0.001)	ηp^2^ = 0.12 ηp^2^ = 0.27

Marusiak, 2019 [41]	RCT	20	PD	73 ± 9.5	55	NR	Cycle ergometer aerobic interval training vs. control (usual care including conventional physical therapy)	S: 8 wks F: 3 D: 1h	TMT Stroop	No difference	–

Rios Romenets, 2015 [37]	RCT	33	PD	63.8 ± 9	24	MoCA 26.9 ± 2.6	Traditional Argentine tango class vs. Control group (usual physical activities and exercise program)	S: 12 wks F: 2 D: 1h	MoCA	No difference	–

Haas, 2024 [38]	Three-arm RCT	94	PD	68.7 ± 9.7	35	Brazilian version of the MoCA 23.0 ± 1	Nordic Walking group vs. Brazilian Dance vs. Deep-water exercise	S: 12 wks F: 2 D: 60 min	Brazilian version of the MoCA	No difference	–

Inskip 2022 [42]	NRCT crossover	9	LBD (*n* = 4) and PD dementia (*n* = 5)	74	22	MMSE 22	Multi-component exercise	S : 8 wks F: 3 D: 60 min	MMSE PD-CRS	+ 4.5 pts (*p* = 0.03) + 8 pts (*p* = 0.03)	Cohen’s *d* 0.32 Cohen’s *d* 0.15

⁎ Square-stepping exercise: a specific training program. The participants were instructed to perform a sequence of different steps on a thin mat (100 £ 250 cm) with 40 squares. The exercise session consisted of walking from one side of the mat to another according to the step patterns. There were sixty-four step patterns performed in the training program.

⁎⁎ goal-based exercise: a standardized exercise protocol that involved walking exercises coordinating upper and lower limbs on a simultaneous or alternating manner, non-progressive muscle-toning exercises using resistance bands and the person’s own body weight, and whole body stretching exercises. BNT: Boston Naming Test; CVLT: California Verbal Learning Test; FAB-it: Frontal Assessment Battery-Italian version; IPBLOT: Intersecting Pentagons and the Benton Line Orientation Test; LBD: Lewy Body dementia; M test: memory with interference test; MMSE: Mini-mental State Exam score; MoCA: Montreal cognitive assessment; NIH Toolbox: National Institute of Health toolbox cognition battery; NR: Not reported; NRCT: non-randomized control trial; PD: Parkinson's disease; RCT: Randomized control trial; Rey-O: Rey-Osterrieth Complex Figure Test; SD: standard deviation; TMT: Trail Making Test.

### 3.3 Physical interventions

The physical interventions were also heterogeneous and comprised aerobic training, resistance training, flexibility training as well as “multicomponent exercise” (**See details in** Tables 1**,** 2 **and** 3). In 9 studies, the physical activity intervention became progressively more difficult and complex. Three studies delivered the interventions individually, sixteen through group sessions, one study employed both modes of delivery in different groups and one study did not report this information. Two studies promoted exercise treatment via home-based telerehabilitation [25,27]. Eleven trials assessed exercise tolerance and adverse events were reported in eight out of ten trials (e.g. knee pain, falls, chest pain, disorientation).

### 3.4 Cognitive outcomes

#### 3.4.1 Alzheimer’s disease

Seven articles involved patients with AD. Table 1 summarizes the detailed characteristics of all the selected articles. In five studies, the diagnosis of AD was based on explicit clinical criteria [[43], [44], [45]] while two did not specify the diagnostic criteria used. Unfortunately, the investigators did not systematically verify the AD diagnosis using biomarker confirmation. The Clinical Dementia Rating (CDR) scale ranged between 0.5 and 2 i.e. from MCI to moderate dementia stages. A total of 3 out of 7 studies, with a limited number of participants, showed significant cognitive improvement on at least one scale after physical activity compared with the control group. Lok et al. included patients with a baseline MMSE score of approximatively 23 [23]. They compared a 12-week intervention of walking and musical exercise to a control group receiving usual care, demonstrating that the MMSE score was significantly higher in the intervention group (mean change = 2.1 points) than in the control group following the exercise period. Holthoff et al. included patients with a mean MMSE of 20.6 ± 6.5 points at baseline and a CDR stage between 1 and 2 [25]. They compared a 12-week leg training intervention to usual care and found that reaction time and motor skills (Fetz test, meters) improved significantly in the experimental group after the intervention, but not the MMSE score. Additionally, executive function and language ability (measured by semantic word fluency and number of words) were significantly better in the intervention group three months post-intervention. Öhman et al. studied two types of intervention comprising customized home-based exercise (*n* = 70) and group-based exercise (*n* = 70), each conducted twice a week for one year, compared to a control group (*n* = 70) receiving usual community care [27]. Executive function, measured using the CDT, improved in the home-based exercise group with changes in the score significantly better than those in the control group at 12 months. However, no significant between-groups differences were found at the 12-month follow-up for MMSE scores. At baseline, Yu et al. involved patients with a mean MMSE of 21.4 ± 3.3 and an ADAS-Cog of 18.8 ± 7.1. They compared the effect of cycling (intervention group) versus stretching (control group) on the ADAS-Cog at 6 and 12 months, finding no significant differences between the groups. The 12-months change in ADAS-Cog for both groups was of approximately 2.5 ± 5.5 points [28]. Lam et al. compared four 6-week programs: physical exercise, working memory training, combined working memory and physical exercise, and health education [22]. Patients had an MMSE of 21 at baseline and after the 6-week training program, all groups showed improvements in the CDR-SOB, verbal fluency test, and delayed recall but not in the ADAS-Cog score. However, there was no significant between-group difference. Hoffmann et al. [24] studied a 16-week intervention of supervised moderate-to-high intensity aerobic exercise (strength, bicycle, cross trainer and treadmill) compared to a control group with usual treatment. The mean baseline MMSE was 24, and a significant effect was found in the change from baseline on the Symbol Digit Modalities Test (SDMT), which assesses mental speed and attention, in subjects who adhered to the protocol, compared to the control group. Finally, the study conducted by Morris et al. [26] assessing the impact of aerobic exercise vs stretching dit not find any significant cognitive change on a wide range of cognitive scales.

#### 3.4.2 Vascular cognitive impairment

Three studies involved patients with vascular impairment including one with stroke and two with subcortical ischemic VCI (*n* = 2) [[29], [30], [31]]. Table 2 summarizes the detailed characteristics of each selected article. Two of the three studies showed statistically significant cognitive improvements associated with the physical activity intervention compared to the control group, despite methodological precautions that should be considered when interpreting the results. In the study by Ihle-Hansen et al., the diagnostic criteria for stroke were not clearly explicated [29]. Included participants had experienced a first or recurrent stroke due to infarction or intracerebral haemorrhage were community-dwelling, with a modified Rankin Scale (mRS) score below 5 and an MMSE score > 21. The 380 patients included had a mean National Institute of Health Stroke Scale (NIHSS) score of 1.6 ± 2.4, a mean mRS score of 1.4 ± 1.1 and a mean MMSE score of 28 ± 0.2. In the two other studies included (Barha et al. and Hsu et al.), the diagnosis of subcortical ischemic VCI was based on usual research criteria for subcortical vascular dementia in clinical trials [46] with a MoCA score < 26 and a MMSE score ≥ 20. Ihle-Hansen et al. investigated the effectiveness of an individualized post-stroke physical activity and exercise intervention program on cognitive function, compared with usual care. The intervention involved regular coaching to perform 30 min of physical activity daily, along with 45–60 min of physical exercise at 2–3 bouts of vigorous intensity per week. No clinically relevant effect of this program was found on cognitive outcomes (MMSE, TMT A and B) after 18 months compared with usual care [29]. The study by Barha et al. examined the impact of aerobic training (walking) for 6 months compared to usual care plus education. Their results showed a significant improvement in TMT A and B performance among female participants, but not males, after six months of aerobic training. However, the study's overall findings were negative, and we emphasize the lack of change in other executive function tests, such as the Stroop test and the digit span test [30]. Hsu et al. showed that 6-month aerobic training in older adults with mild subcortical ischemic VCI significantly improved flanker task reaction time, after adjusting for baseline general cognition, total white matter lesion volume and flanker performance [31].

#### 3.4.3 Parkinson’s disease

Ten articles involved patients with Parkinson’s disease [[32], [33], [34], [35], [36], [37], [38], [39], [40], [41]]. Table 3 summarizes the detailed characteristics of the selected studies. The diagnosis was based on the UK Brain Bank Criteria [47] in 3 studies [35,36,39] and on the London Brain Bank Criteria [48] in one study [38]. In the other six studies, the diagnosis of PD was based on clinical assessment, with no further details on the criteria applied. The mean age of patients ranged from 62.3 to 74 years. The mean UPDRS motor subsection score ranged from 15.1 ± 1.1 to 48.9 ± 11, with three studies indicating no motor score. Six studies reported the Hoehn and Yahr stage of diagnosis: three studies included patients with stages ranging from 1 to 3, one study included patients between stages 1.5 and 3, one between stages 1 and 2.5, and one study included patients at stage 3. Additionally, four studies did not report the Hoehn and Yahr score as part of the inclusion criteria. The mean Hoehn and Yahr staging scale ranged from 1.6 ± 0.8 to 3.2 ± 0.4 (information was missing in two studies). Six studies included dementia-free patients with MMSE scores above 26 in one study, above 24 in 2 studies and above 23 in one study, and with MoCA scores above 21 in one study, and with ACE scores above 82 in another study. Cognitive status was not regarded as a strict inclusion criterion in the other studies, but patients had to be cognitively able to follow a physical activity program. Of the ten studies, four reported a significant improvement in cognitive performance as a function of the physical activity tested. Kalyani et al. showed that dance had a significantly positive impact on auditory verbal learning test and TMT-B in 19 patients with PD [33]. Picelli et al. showed that 4 weeks of treadmill training led to significant improvement for Frontal Assessment Battery-Italian version, TMT A and B and memory with interference between groups after intervention but not for MoCA [35].

#### 3.4.4 Lewy body and Parkinson’s disease dementia

One study involved 9 patients having LBD (*n* = 4) or PD dementia (*n* = 5) [43]. The characteristics of the study are also detailed in Table 3. No further information on diagnostic criteria was found [49]. The mean age of patients was 74 years, 23 % were female, and the mean MMSE score at baseline was 22. In a non-randomised, non-blinded, crossover trial involving a baseline assessment, an 8-week wait-list, and an 8-week exercise intervention, Inskip et al. found that multicomponent exercise intervention (static balance, dynamic balance, functional practice and high intensity progressive resistance training) resulted in significant improvement of cognition. After 8-week wait-list, decline in cognition as measured by the MMSE (*p* = 0.06) and PD-CRS (*p* = 0.67) was not significant. After 8-week exercise intervention, cognition improved significantly in all participants for the MMSE (*p* = 0.03) and PD-CRS (*p* = 0.03), and two participants improved beyond the minimal clinically important difference for PD-CRS.

## 4 Discussion

Research in the field of cognition and physical exercise has experienced significant growth in recent years, and this literature review specifically emphasized the benefits of physical activity within groups of patients suffering from a given neurocognitive pathology. In general, the conclusions of the published studies indicate beneficial effects in older patients with cognitive disorders (14 studies out of 21). Engaging in aerobic exercise, either by itself or as part of a comprehensive program that includes strength and balance training, was suggested to help improve or maintain cognitive performance or physical functional capacity [50]. The results were inconsistent among studies and the magnitude of the effect was modest. First, in the studies involving participants with AD, we found that exercise-related increase in cardiorespiratory fitness was related to improvement in memory performance and reduced hippocampal atrophy [26]. In Öhman *et al*. study only home-based exercise (but not group exercise) was associated with improvement in executive function, but not in global cognition or memory [27]. Holthoff et al. found small improvements in language and executive functions but not global cognition after only 12 weeks [25]. Lok et al. reported small improvement in global cognition and mood after 12 weeks [23]. Our results highlighted a dose-response relationship with physical activity, as the negative studies [24,28] involved relatively low weekly exercise levels (100 to 150 min), while the others included up to 200 min per week. Several hypotheses may explain these results, based on biological effects targeting AD lesions. Physical activity may lower both β-amyloid and brain tau burden in older adults, even though these findings have only been described in cognitively unimpaired participants [51,52]. In genetically driven early-onset autosomal dominant AD, mutation carriers who performed more than 150 min/week of physical activity showed both less CSF AD lesions and better cognitive and functional performance than those who performed low physical activity [53]. Greater physical activity could also mitigate the negative association between β-amyloid burden and neurodegeneration in cognitively unimpaired individuals [54]. Another possible explanation would be benefits on brain metabolism related to physical exercise: better glucose absorption [55] or improved mitochondrial function [56]. Six-month resistance training was efficient on global cognition in adults with MCI, without specific effect on memory. The authors suggested a potential role of increase in IGF-1 or reduction in homocysteine and inflammation [57]. All these factors play a role in the pathophysiology of AD. PD is the second most common neurodegenerative disease, but in this review, it represents the primary condition studied to evaluate the benefits of physical activity on cognitive function. Exercise has always been important in PD to improve motor and non-motor symptoms, which constitute the primary endpoints in most studies evaluating physical activity in these subjects [58]. A clinical trial, which was designed to investigate the dose-response of treadmill exercise to PD, showed that only high-intensity exercise successfully improved motor symptoms of PD patients [59]. Nonetheless, cognitive benefits related to physical activity can be expected in this context. It seems that the benefits of physical activity could be mediated by dopamine, as there is a relationship between global cognition and dopamine transporter availability in PD which is mediated by physical activity [60]. Surprisingly, we did not find any studies dedicated to LBD except for the study by Inskip et al. involving only 4 subjects with LBD among other Parkinson's patients, suggesting cognitive benefits from training sessions combining static balance, dynamic balance, functional practice, and progressive resistive exercise. To be noted, cognitive stimulation may conversely increase the level of physical activity in adults with MCI due to PD [61]. Regarding VCI, we included two studies which similar designs (walking 3 times a week for 60 min over 6 months) reporting inconsistent cognitive benefits associated with physical activity [30,31]. On the other hand, regular coaching to encourage physical exercise in stroke patients was not associated with cognitive improvement [29]. Barha et al. highlighted an interesting increase in brain derived neurotrophic factor, which was associated with cognitive improvement in women only. However, changes in cardiovascular risk factors, particularly blood pressure, were not monitored in these studies. In patients with atrial fibrillation regular exercise was associated with better cognition as well as lower prevalence of ischemic infarcts and of moderate to severe white matter lesions [62]. The benefits of exercise in VCI may also be mediated by increased brain oxygen supply due to increased blood flow [63]. Overall, physical activity may improve cognitive function in adults over 50, regardless of their cognitive status. Patients should engage in both aerobic and resistance exercise of at least moderate intensity as many days of the week as possible [64]. Physical activity is also recognized as an epigenetic modulator of brain plasticity and cognition, with evidence showing its general cognitive benefits independent of specific pathologies [65]. Studies have also suggested that physical exercise may offer cognitive benefits in patients with MCI or dementia of various causes [[10], [11], [12]]. In general, the level of physical activity was quantitatively associated with brain lesions [66]; however, the effect size observed in the studies included in our review appears modest. Various factors, such as exercise intensity, duration, and training mode, may mediate and moderate these effects. Aerobic and resistance exercise training have been suggested as the best interventions for enhancing cognitive functions [67]. Physical activity also offer non-cognitive benefits such as mood improvement, which may indirectly affect cognitive function. Group physical activity programs help reduce social isolation, thereby enhancing patients' moods. Beyond cognitive advantages, physical activity also improves gait quality, aids in fall prevention, and enhances overall quality of life [68]. By increasing participants’ physical functioning and independence, this intervention supports the recommendation that individuals with AD engage in regular, supervised home-based physical activity [27]. Our literature review provides new insights by allowing us to analyze the short-term effects of physical activity on cognitive functions in individuals with neurological diseases. However, understanding the long-term effects of such interventions, as well as the persistence of benefits after discontinuing physical exercise, remains challenging and unclear. Only three studies had a follow-up of more than 12 months and showed an improvement in cognitive outcome [27,29,39]. It is noteworthy that among sedentary older adults, a 24-month moderate-intensity physical activity program, when compared to a health education program, did not result in improvements in global or domain-specific cognitive function [69]. Further research with long longitudinal follow-up is needed to clarify this issue. The sex ratio varied across studies and could influence the effect of physical activity on cognition. Biological differences between men and women shape their responses to physical activity and their susceptibilities to the onset, progression, and outcomes of neurodegenerative diseases [70]. Incorporating biological sex into analyses of the neurobiological effects of physical activity provides deeper insight into the mechanisms that promote brain health. Additionally, socioeconomic status—often overlooked in these studies—represents a significant confounding factor. It can affect adherence to physical activity programs, thereby modulating the associated cognitive benefits. Many studies have examined the impact of multidomain interventions—combining physical activity, dietary changes, and cognitive stimulation—to prevent cognitive decline in individuals at risk of dementia [71]. However, these studies were not designed to evaluate the individual effects of each intervention. It is plausible that these combined approaches may enhance the overall impact on cognition. Our literature review highlights that physical activity plays a crucial role in the success of such interventions, underscoring its importance as one of the most effective strategies for maintaining cognitive health. The main strength of our study was the rigorous selection of published studies to investigate the benefits of physical exercise in individuals selected by pathology. However, our review has several limitations that must be acknowledged. Many studies relied on multidomain interventions, such as combining physical activity with other treatments (e.g., therapeutic education or nutrition), making it difficult to isolate the specific effects of adapted physical activity. Additionally, the number of participants varied across studies, as did the control groups in terms of both number and type. The number needed to treat was not systematically reported in the included studies, preventing a comprehensive assessment of the clinical significance of the interventions. Most studies also lacked blinding in the interventions, which could introduce bias. Furthermore, important factors that may influence the effects of physical activity, such as comorbidities or body composition, were often overlooked in these assessments. Confounding factors such as age, biological sex and socio-economic status varied from study to study. All the studies included in this review diagnosed AD based on clinical criteria without biomarker confirmation, hence a risk of over-classifying patients as having VCI. We have previously highlighted the heterogeneity in the published literature, including variations in diagnostic criteria, relatively short intervention durations, and the lack of biological measurements that might explain individual responses to physical activity. In addition, we cannot exclude the possibility of publication bias, given the growing number of studies in this field. Finally, the heterogeneity of the populations, type and duration of interventions and cognitive outcomes did not allow us to carry out a meta-analysis to identify the best physical activity strategies to adopt.

## 5 Conclusions and perspectives

In conclusion, physical activity stands out as one of the most promising non-pharmacological interventions for improving cognitive function in patients with neurodegenerative diseases and VCI. Although findings vary considerably across studies—likely due to differences in the type of physical activity prescribed and the evaluation criteria used—some evidence suggests potential cognitive benefits. These findings are in line with recent international consensus recommendations supporting the implementation of tailored physical activity programs in patients with MCI or dementia to help preserve cognitive and functional abilities, despite the current limitations in high-quality evidence [72]. Exercise also provides substantial overall health benefits, including improvements in motor symptoms, physical function, and quality of life, which are often compromised in neurodegenerative diseases. The absence of significant side effects further supports its inclusion as a key component in therapeutic strategies. To optimize the benefits, patient care must be individualized, taking into account specific clinical presentations. This literature review highlights the heterogeneity of populations, interventions, and cognitive outcomes in this field of research. Standardizing evaluation criteria and implementing longitudinal follow-up in future studies will be critical in more accurately assessing and maximizing the impact of physical activity on both cognitive and general health outcomes.

## Funding

None.

## CRediT authorship contribution statement

**Théodore Decaix:** Writing – review & editing, Writing – original draft, Visualization, Supervision, Methodology, Formal analysis, Data curation, Conceptualization. **Claire Bonnin:** Writing – original draft, Methodology, Formal analysis, Data curation, Conceptualization. **Karl Götze:** Writing – review & editing. **Véronique François:** Writing – review & editing. **Camille Petit:** Writing – review & editing. **Clémentine Rivière:** Writing – review & editing. **Sandrine Greffard:** Writing – review & editing. **Emmanuel Cognat:** Writing – review & editing. **Jacques Hugon:** Writing – review & editing. **Claire Paquet:** Writing – review & editing, Supervision. **Louise Sindzingre:** Writing – review & editing, Writing – original draft, Formal analysis, Data curation. **Matthieu Lilamand:** Writing – review & editing, Writing – original draft, Validation, Supervision, Methodology, Formal analysis, Data curation, Conceptualization.

## Declaration of competing interest

The authors declare that they have no known competing financial interests or personal relationships that could have appeared to influence the work reported in this paper.
